# Rapid Robot-Aided On-site Testing of Fume Cupboards

**DOI:** 10.1093/annweh/wxac043

**Published:** 2022-06-18

**Authors:** Matthias Bittner, Claude Moirandat

**Affiliations:** SKAN AG Switzerland, Kreuzstrasse 5, 4123 Allschwil, Switzerland; CMD, Blotzheimerstrasse 19a, 4055 Basel, Switzerland

**Keywords:** containment test, dummy, fume cupboard, IPA isopropanol, on-site test, robotic

## Abstract

Although containment testing of fume cupboards (FC) according to the standards EN 14175-3 (2019) or ANSI/ASHRAE 110 (2016) is well established for type testing, its application is currently much less accepted and practised for evaluating containment on-site. Few of the several million FC in the market have been tested at installation and commissioning, and even less undergo verification of containment during their service life in the laboratories. Several reasons have led to this unsafe situation. To address this challenge, a new concept has been developed to allow for rapid on-site testing of FC to gain knowledge as to the functional efficiency as well as to safety aspects for the operator. The concept consists of a movable robot-aided test equipment that can be installed quickly to the FC in running labs. Multiple sensors detect the tracer gas isopropanol. Within a test run of only 10-min data is collected to quantify containment at the sash opening and to determine purge efficiency. The method reveals impact from interfering effects such as draughts, air distribution, and movements and from equipment installed, and is a tool for the optimization of operating conditions of a lab. This article presents an advanced alternative to the existing containment tests, particularly for on-site testing. The method assesses not only proper operation of the FC in its environment, but also the suitability of a FC for a given use under aspects of health and safety evaluation.

**What’s important about this paper?**

While there are well-established methods for testing fume cupboards, few cupboards are tested for containment at installation, and fewer are tested over their service life. To address this challenge, a new approach has been developed to allow for a rapid on-site testing of fume cupboard containment effectiveness that involves movable robot-aided test equipment that can be quickly deployed in operating laboratories. This study describes this approach and demonstrates its utility.

## Introduction

Fume cupboards (FCs) or laboratory fume hoods ‘are perhaps the most widely used and misused safety devices’ (SEFA-1 Recommended Practices 1, 2010). Worldwide several millions of operators rely on the correct performance of FC to protect the lab personnel from potentially hazardous airborne contaminants. Manufacturer design and test FC according to current standards as per EN 14175-3 (2019) or ANSI/ASHRAE 110 (2016). These tests are done to characterize the FC and its containment from a manufacturer’s point of view. These include a spacious test room, conditioned air supply, in the absence of draughts, personnel, and installations. Only one procedure of the EN 14175-3, the robustness test, creates artificial draughts in front of the FC using a moving plate as disturbance factor.

Once installed in a lab, FCs are in a totally different and challenging environment: many FCs and hoods are competing with each other on the exhaust air system, for example. A huge volume of supply air must find its way into these devices and then as extract air out of the lab. Personnel walk by and work at the FC using equipment and chemicals within the workspace. All these elements influence the containment of an FC and consequently the protection of the personnel from exposure to contaminants. Type tests as described in the standards to date do not reflect the lab situation, including ASHRAE 110 (Mattox *et al*., 2019) and EN 14175 (Sieber, 2012), and therefore their value for assessing field performance is disputable. Reference values obtained in such tests, often single p.p.m. values only, are figures unrelated to reality and hard to interpret. The results of the qualification do not reflect the real FC use, neither the chemical load nor the risk potential. From today’s perspective, an evolution of containment testing on-site would be beneficial to improve the relevance for the operator’s health and safety.

## Current tracer gasses and limitations associated

Current standards for FCs have a long history and their methodology goes back to the 80s. Growing constraints are related to environmental and technical aspects:

- The tracer used to date, sulphur hexafluoride (SF_6_), is classified as a potent greenhouse gas with the highest global warming potential (GWP_100_) of 22 800 kg CO_2_ per kg of SF_6_ (Code of Federal Regulations, 202; Global Warming Potentials, 40 CFR Part 98 Subpart A, Table A-1). Some countries have already banned its use or are restricting it to a few applications. Environmental policies and exemption permit therefore complicate its handling (Chemical Risk Reduction Ordinance, Swiss Confederation, 2022).
- Laughing gas or nitrous oxide (N_2_O) has been proposed as a substitute for SF_6_. Few examinations have shown its suitability as a tracer for this application, such as those described by Burke *et al.* (2014). It has still a high GWP_100_ of 298 kg CO_2_ per kg of N_2_O. In addition to environmental concerns, health risks and the abuse of N_2_O as a party drug led to growing objections over its use as for FC testing (Marcus, 2021).
- Most analytical instrumentation used to detect and quantify SF_6_ are IR photometers. Their measuring principle requires tracers to be absent in the room air, and only tracers with a high heat absorption satisfy selectivity and sensitivity requirements. Suitable tracers therefore inherently have high GWP_100_ values (Code of Federal Regulations, 2022).
- The standard containment test setups for sampling do not allow localizing breakouts over the sash opening. The samples in the outer grid or robustness test as per EN 14175-3 are pooled in a single photometer diluting and blurring the sample information. The inner grid test asks for sequential sampling to evaluate the sash opening as does the testing according to ASHRAE 110.

Thus, besides the limited approach to the practical FC use and lab reality, the methodology of the standards suffers from additional constraints. These built the target for the development of an alternative test procedure and hardware to analyse more deeply the safety conditions for the user in his real lab environment.

## Robot-aided containment test equipment

**Figure 1.**
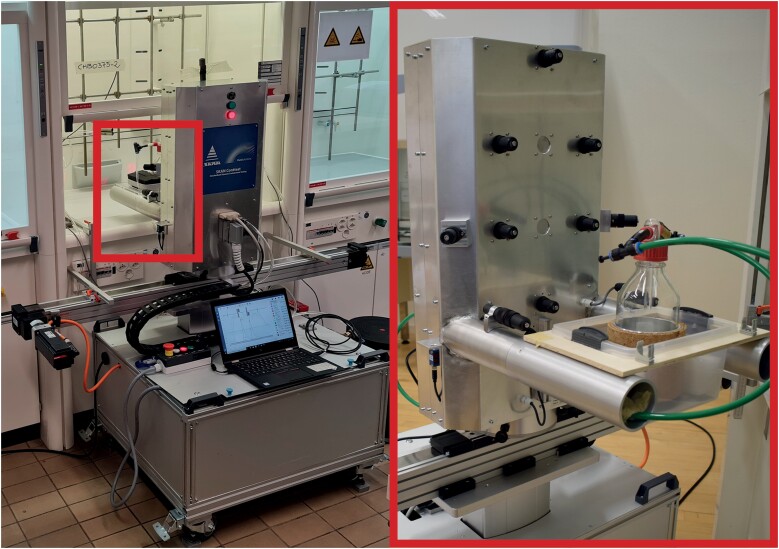
A Conttest test setup under field conditions (photograph by courtesy of EPFL, CH-Lausanne), at left. View on front side of the body with sensors (black cartridges on the body), arms, and vaporizing bottle, at right.

The test concept developed, named ‘Conttest’, as the abbreviation of ‘containment testing’, is a holistic approach to overcome the limitations. The hardware is shown in Fig. 1 in a typical test situation in the lab.

A compact movable base unit is installed in front of the FC’s work opening with the sash opened to a test height of 0.5 m ± 1% according to EN 14175-3 (2019). The base unit carries a movable dummy body (dimensions W 0.4 m × H 0.7 m × D 0.2 m). The robot dummy moves horizontally on a linear drive bar to the right and left at a constant speed of 0.4 m/s following a standardized sequence. This setup mimics an operator working in the sash opening disturbing the inflow pattern to reflect the practical situation. Light sensors detect the track end points, supporting the installation to the FC. Another drive allows for placing the dummy at correct height respective to the work surface. The arms of the dummy extend 0.3 m through the sash opening (2) into the workspace at 0.05 m above work surface. They hold a glass bottle as obstruction.

Ten semiconductor sensors (MOS from SM Elektronik, PN. 850-115) are attached to the dummy. These sensors sample the local tracer gas concentrations near the dummy’s body. The distance from the dummy’s sensor plane to the sash plane is 0.1 m. The uppermost sensor on the dummy (height over ground ~1.5 m) samples the operator’s breathing zone. Its height above the work surface is 0.58 m. Two additional sensors are placed inside the FC to continuously monitor the challenging concentration as positive control. The sensors, designed for a recurring calibration, have a sensitivity ranging from 0.2 to 300 p.p.m. for IPA delivering signals with a response time of 4 s.

Gaseous isopropanol (IPA) is generated from a ≥99.5% liquid by means of a forced air flow. IPA is an environmentally friendly alcohol widely used as a lab chemical. It does not create residuals. It has been applied also within the ASHRAE research project RP 1573, described by Smith (2020) as a potential alternative to SF_6_. The IPA tracer gas is released through six ejector tubes fitted on two stands left and right within the workspace. The tracer flows at a constant rate and directed towards the front opening and the dummy as a challenge with momentum. The IPA emitted in the workspace creates mean concentrations of 50–100 p.p.m., with peaks reaching 1000 p.p.m., and is safe in respect to exposure limit values (Suva, 2022) for operators.

A controller drives all components to run through a test cycle. Data are transmitted to a logger and displayed in real time on a monitor to be further processed, statistically analysed and compiled readily into a test report. The principle of the Conttest method and its application is shown in an animation video accessible on the internet (Bittner, 2021).

## Presentation of results with the new method

The research work was conducted partly on-site under field conditions and partly on a standardized bench top FC [used by European type test labs (1.5 m width) for evaluation purposes] installed in a large lab room without climate control, just ventilated by the FC.

**Figure 2.**
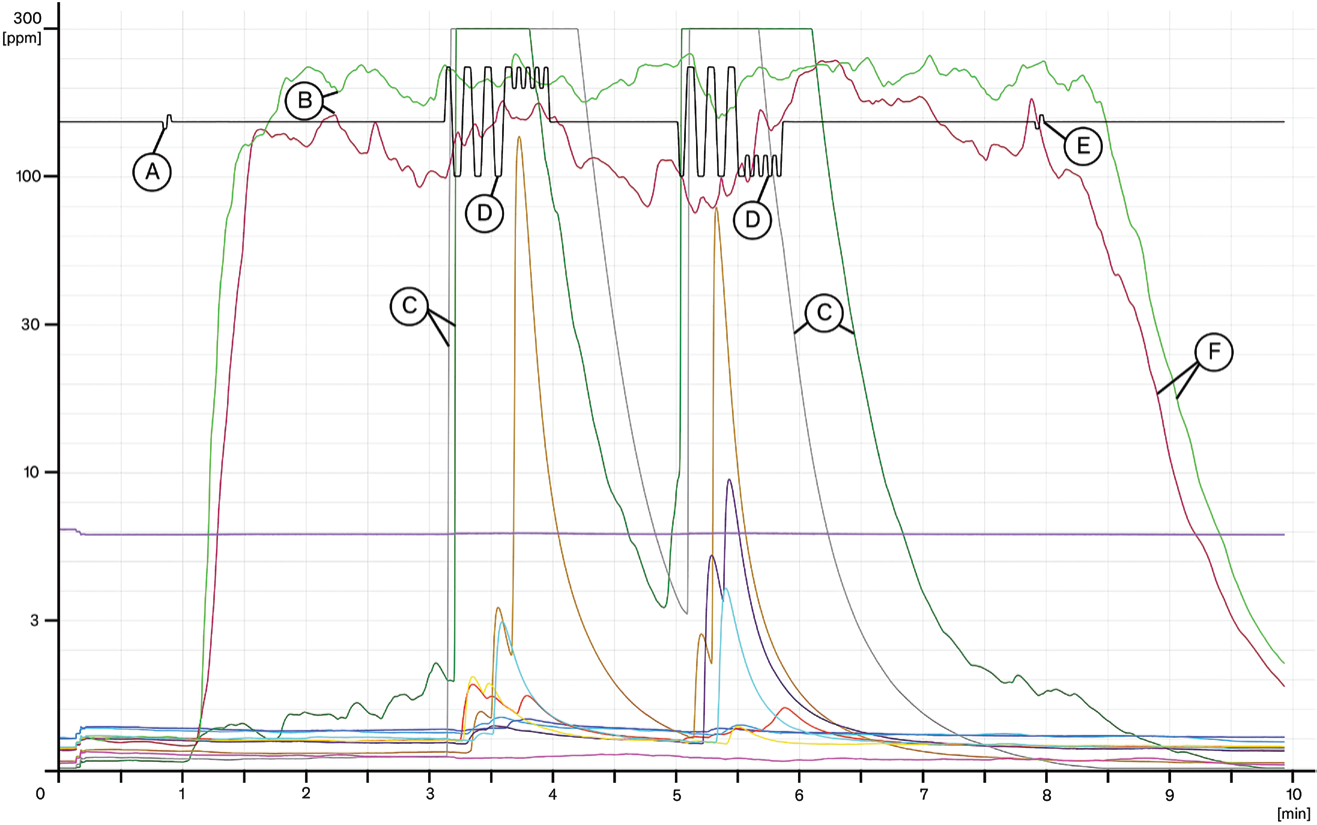
Conttest diagram (raw data) with a bench-type FC (width 1.5 m) with a box (0.27 × 0.36 × 0.28 m) as obstacle placed in the right rear corner of the FC running at 500 m^3^/h extract air volume flow rate. The asymmetry of the body sensors during the two movement phases results from the obstacle. For definitions A–F, see text.

A graph of a raw data print is shown in Fig. 2. The *x*-axis is the timeline, the *y*-axis the concentration of tracer gas on a logarithmic scale from 1 to 300 p.p.m. IPA. The signals of 12 sensors are recorded individually, shown by coloured lines. The black line documents the body movement. A small spike (A) indicates the tracer gas being switched on. The internal tracer gas concentration is displayed with two curves for the right and left sensor inside the workspace (B) reaching ~100 p.p.m. during the exposure. The sensors on the dummy body show various peaks, whereas the two arm sensors (C) reveal strong exposure during movement of the body (D). Near the end of the procedure, the gas is switched off (E), and the concentration inside the workspace is decaying due to continuous dilution by make-up air (‘purging’, F). The gradient of the purge curve is a function of the extract volume flow rate and a characteristic of each FC.

Effects from installations left in the FC without clearing the workspace for containment testing are included when identifying unsafe situations. The mapping with 12 sensors within a single test run allows to localize impacts from FC design and interferences such as air flows, obstacles, or draughts. Performed on-site, this procedure reflects actual conditions of use and reveals optimization potential for tuning air volumes, sound level, and energy consumption. The concept is applicable to recirculatory filter fume cabinets as the active carbon filter media adsorbs the IPA.

Further tests with obstacles or disturbances confirmed the capability of the Conttest method to recognize influences on containment and were conducted in collaboration with the test house FC^2^S Tintschl. The pattern as in Fig. 2 indicates multiple differences to the pattern without obstacles. The sensors detect outbreak at a higher rate, contrary to the robustness test results as per EN 14175-3, which even implicate a better containment. The sample grid of the robustness test with just one sensor signal and a single p.p.m. value loses resolving power to identify the disturbing effect. Evaluation with multiple sensors and discrete signals enables a mapping to characterize an FC, building the base for a subsequent analysis, e.g., for an occupational health risk assessment.

## Conclusion

The exposure of the operator to emissions under real lab conditions, including movement, interfacing, obstructions, and draughts, is essential for qualifying the containment of fume cupboards. The concept presented here considers these aspects to assess containment on-site. It utilizes isopropanol gas as alternative to SF_6_ combined with a differentiated detection of the tracer. The multiple sensors array delivers a ‘finger-print’ pattern of a FC’s behaviour as to containment under work conditions. Test data are presented as comprehensible diagram delivering figurative information applicable for an assessment on health and safety evaluations. The concept overcomes known weaknesses of current standard test procedures and provides a quick setup on-site. It contributes to the optimization of running lab conditions.

## Data Availability

Supplementary data underlying this article will be shared on reasonable request to the corresponding author.
